# Large‐scale analysis of BAP1 expression reveals novel associations with clinical and molecular features of malignant pleural mesothelioma

**DOI:** 10.1002/path.5551

**Published:** 2020-10-15

**Authors:** Assunta De Rienzo, Lucian R Chirieac, Yin P Hung, David T Severson, Samuel Freyaldenhoven, Corinne E Gustafson, Nhien T Dao, Claire V Meyerovitz, Michela E Oster, Roderick V Jensen, Beow Y Yeap, Raphael Bueno, William G Richards

**Affiliations:** ^1^ The Thoracic Surgery Oncology Laboratory and the International Mesothelioma Program (www.impmeso.org), Division of Thoracic Surgery and the Lung Center Brigham and Women's Hospital, and Harvard Medical School Boston MA USA; ^2^ Department of Pathology Brigham and Women's Hospital, and Harvard Medical School Boston MA USA; ^3^ Department of Pathology Massachusetts General Hospital and Harvard Medical School Boston MA USA; ^4^ Department of Biological Sciences Virginia Tech Blacksburg VA USA; ^5^ Department of Medicine Massachusetts General Hospital and Harvard Medical School Boston MA USA

**Keywords:** mesothelioma, BAP1, immunohistochemistry, tumor suppressor gene, intra‐tumor heterogeneity, epithelial‐to‐mesenchymal transition, gene expression, prognostic biomarker

## Abstract

*BRCA1‐associated protein‐1* (*BAP1*) expression is commonly lost in several tumors including malignant pleural mesothelioma (MPM). Presence or absence of immunohistochemical BAP1 nuclear staining in tumor cells is currently used for differential diagnosis of MPM. In this study, a large cohort of 596 MPM tumors with available clinical data was analyzed to examine associations of BAP1 staining pattern with clinical and molecular features that may reflect the impact of *BAP1* mutation on MPM biology. Cases were classified according to the BAP1 staining pattern of tumor cells. Exome and RNA‐sequencing data were available for subsets of cases. Levels of mRNA encoding claudin 15 (*CLDN15*) and vimentin (*VIM*) were determined using RT‐qPCR on 483 cases to estimate the relative proportions of epithelial‐like and mesenchymal‐like components in each tumor. Four BAP1 staining patterns were observed: single‐pattern nuclear staining (36%), single‐pattern cytoplasmic staining (25%), single‐pattern absent staining (12%), and combinations of these staining patterns (27%). This study confirmed prior reports that nuclear BAP1 is more frequently associated with wild‐type *BAP1* and sarcomatoid histology. However, no associations between BAP1 staining pattern(s) and mutations in specific protein domains and/or mutation type were observed. BAP1 staining patterns were significantly associated (*p* < 0.001) with *BAP1* gene expression, MPM histologic subtypes, molecular clusters, and markers of epithelial‐to‐mesenchymal transition. Frequent observation of combinations of BAP1 staining patterns in MPM tumors indicated intra‐tumoral heterogeneity of *BAP1* status. Cytoplasmic BAP1 staining was identified as a putative indicator of favorable prognosis in non‐epithelioid MPM. In conclusion, novel significant associations among different BAP1 staining patterns and subgroups of MPM tumors were observed, suggesting that the role of *BAP1* in tumor progression may be more complex than its presumed tumor suppressor function. Cytoplasmic staining was identified as a putative indicator of favorable prognosis in non‐epithelioid MPM, potentially addressing a critical need in clinical decision‐making in this disease. © 2020 The Authors. *The Journal of Pathology* published by John Wiley & Sons, Ltd. on behalf of The Pathological Society of Great Britain and Ireland.

## Introduction

Malignant pleural mesothelioma (MPM) is a rare aggressive tumor arising from the pleura and associated with asbestos exposure affecting 3200 patients annually in the US [1]. The World Health Organization classifies MPM into three histological subtypes: epithelioid, sarcomatoid, and biphasic (comprising both epithelioid and spindle‐shaped cells) [1]. Prognosis is poor for all MPM patients, but those with non‐epithelioid subtypes have a particularly aggressive natural history and do not respond to currently available treatments [1].

*BRCA1‐associated protein‐1* (*BAP1*), located at 3p21.1, a region frequently deleted in MPM, encodes a deubiquitinating enzyme that regulates key cellular pathways [2]. *BAP1* is mutated in up to 60% of MPM samples, including germline mutations [3, 4, 5]. However, the impact of *BAP1* mutation on MPM biology remains poorly understood. BAP1 protein consists of 729 amino acids and contains several domains including binding regions for protein interaction partners, and nuclear localization signal (NLS) targeting motifs required for nuclear localization [6].

*BAP1* has garnered both research and clinical focus in MPM based on its high rate of mutation, demonstrated role in other malignancies [2], and common use as an immunohistochemical biomarker for MPM diagnosis, particularly for the epithelioid subtype [7, 8, 9, 10, 11, 12, 13, 14, 15, 16, 17, 18, 19, 20, 21, 22, 23, 24]. The monoclonal antibody C4 (Santa Cruz Biotechnology, Dallas, TX, USA), utilized in most clinical and investigational settings [3, 5, 7, 8, 9, 10, 11, 12, 15, 16, 17, 18, 19, 20, 21, 22, 23, 25, 26, 27], binds within the C‐terminal region of the BAP1 protein (aa 430–729). Clinical and research use involves binary evaluation of presence or absence of BAP1 nuclear staining, defined as positive when the nuclei of tumor cells exhibit immunoreactivity. Presence of nuclear staining has been associated with wild‐type *BAP1*, whereas complete absence of cellular staining correlates with *BAP1* biallelic loss [3, 17, 21]. Nuclear BAP1 staining is more common in sarcomatoid disease [11, 15, 18], consistent with studies describing biphasic cases with BAP1 nuclear positivity in the sarcomatoid component and negativity in the epithelioid component [9, 18, 25]. The significance of cytoplasmic BAP1 immunostaining is thought to represent either a mutation in the ubiquitin C‐terminal hydrolase (UCH) domain [6, 17, 28], deleted/inactivated NLS2 [6, 29], or a non‐specific reaction of the antibody (either classified as *BAP1* loss or removed from the dataset) [10, 11, 12, 13, 14, 18, 25].

Most prior investigations utilized small numbers of samples [13, 14, 15, 22, 23, 25, 27, 30]. Those including larger numbers have used biopsies [7, 10, 11, 14, 17, 27], or tissue microarrays [8, 12, 16, 19], including tumor samples insufficient to evaluate potential intra‐tumoral heterogeneity of BAP1 staining [9, 10, 13, 23, 25]. The objective of the current study was to examine the immunohistochemical localization of BAP1 using clinical blocks from a large cohort of surgically resected MPM cases, of which 263 had *BAP1* sequencing data.

Our primary objectives were to investigate reported associations between loss of nuclear staining and *BAP1* gene mutation [3, 17, 21], cytoplasmic BAP1 staining and mutation in the UCH and/or NLS2 domains [6, 29], and nuclear BAP1 localization and sarcomatoid histology [11, 15, 18]. Exploratory evaluations of the frequency of intra‐tumoral heterogeneity of BAP1 staining, and association of observed staining patterns with clinical, pathological, molecular, and patient outcome variables were also performed.

## Materials and methods

### Clinical samples

Formalin‐fixed and paraffin‐embedded (FFPE) pleural tumor specimens, representing 596 patients diagnosed with MPM, collected between 1989 and 2015 and annotated with Institutional Review Board approval (Partners Protocol: 1999P001980; Dana Farber Cancer Institute Protocol No 98‐063), were retrieved from the Pathology Department archives at the Brigham and Women's Hospital (Boston, MA). Clinical FFPE blocks obtained at surgical resection were selected based on hematoxylin and eosin (H&E) staining to include areas of tumor. To avoid loss of antigenicity, all slides were fresh‐cut and immediately stained in batches of 58 slides. For each block, the first section (15 μm thick) was discarded; the deeper section (5 μm thick) underwent H&E staining; and additional 5‐μm‐thick sections were mounted on charged slides for immunostaining.

### Immunohistochemistry (IHC)

IHC was performed using a monoclonal antibody to BAP1 (clone C‐4; Cat. No. sc‐28383; Santa Cruz Biotechnology, Dallas, TX, USA; 1:100 dilution) using the Leica Biosystems Refine Detection Kit with citrate antigen retrieval. BAP1 nuclear signal in non‐neoplastic cells on the same slides served as an internal positive control. Cytology FFPE blocks prepared from two mesothelioma cell lines, JMN (*BAP1* wild‐type) and H28 (*BAP1* mutated), were included with each batch as external positive and negative staining controls, respectively, because they show presence and absence of BAP1 protein when analyzed by western blotting (data not shown).

The IHC slides were interpreted by one of two pulmonary pathologists (LRC, YPH) who were blinded to case annotation data, and had agreed upon common scoring criteria by analyzing together the first 70 (12%) slides. IHC staining of tumor cells was classified as nuclear, cytoplasmic, absent or combinations of these patterns. Single‐pattern nuclear staining denoted cases for which only a nuclear pattern was present in tumor cells. Single‐pattern cytoplasmic staining was identified in a subset of cases without nuclear staining of tumor cells. Single‐pattern absent staining denoted complete absence of staining in neoplastic cells in the presence of a positive internal control. Combination patterns of BAP1 staining were classified as absent/nuclear, absent/cytoplasmic, nuclear/cytoplasmic, and absent/nuclear/cytoplasmic when the same tumor displayed multiple patterns each representing at least 5% of tumor cells.

### Molecular analysis of BAP1

*BAP1* mutational status was available for 263/596 (44%) tumors from prior deep‐sequencing analyses [4, 31, 32] (see supplementary material, Table S1), including *BAP1* non‐synonymous single nucleotide mutation and indels ≤ 50 nucleotides, referred to in this article as single nucleotide and small variants (SNSVs).

Sequencing data from 164 tumors [4] were reanalyzed by visual inspection of the aligned reads, and mutations in *BAP1* were newly identified in 23 samples. *BAP1* mutational status was available for 101 samples from previous targeted sequencing data [31]. For 73 cases, *BAP1* exome sequencing data obtained in the context of institutional genotyping were retrieved [32]. Eighty‐one cases had been sequenced in more than one analysis (supplementary material, Table S1).

*BAP1* copy number alterations (amplification or deletion > 50 nucleotides) were available for 174/263 cases. Raw counts from RNA‐sequencing data were available for 174 cases [4], and were normalized to transcripts per million (tpm) with gene length and trimmed mean (TMM) scaled library sizes in edgeR. The median expression value for *BAP1* was 13.2, ranging between 1.4 and 52.1 tpm (supplementary material, Table S2).

The availability and overlap of samples included in different subset analyses are shown in supplementary material, Table S3.

### Reanalyzed mutation analysis (RMA)

The exome and targeted sequencing (SPET) reads for each sample included in our previous work [4] were aligned to the *BAP1* gene defined by the Ensembl annotated sequence (ENSG00000163930) for the 8889 bp locus on chromosome 3 downloaded from Ensembl on 21 June 2019. Geneious 11.0.4 (Biomatters, Ltd, Auckland, New Zealand) was used to align the fastq files to the *BAP1* locus using the ‘medium sensitivity’ settings that allow for 30% mismatch and 15% gaps in the read alignments to facilitate identification of large indels. The Geneious ‘Contig View’ alignment displays were visually inspected to identify candidate mutations (SNPs and indels) that were found in *BAP1* coding regions or at splice sites in at least two, non‐identical reads. The RMA results tabulated in supplementary material, Table S1 found approximately 50% more disruptive mutations than previous analyses. The nucleotide positions of the base substitutions, deletions, or insertions in supplementary material, Table S1 are given in the hg19 coordinates for chromosome 3 to be consistent with the prior *Nature Genetics* (NG) [4], *Cancer Research* (CR) [31], and Oncopanel (OP) analyses. The locations of the disrupted amino acids associated with the non‐synonymous mutations, SNPs, FRAMESHIFTs, and loss of exon SPLICE sites are provided relative to the canonical BAP1 protein NP_004647.1 corresponding to the cDNA NM_004656.3.

### CLDN/VIM molecular test

Reverse transcription‐quantitative PCR (RT‐qPCR) was performed to determine the gene expression ratio [33] of *CLDN15*/*VIM* expression (C/V score) [4] in 483/596 cases with available frozen tumor samples, including 169 cases that overlapped with the samples analyzed by RNA sequencing [4]. In brief, RNA was extracted from tumor‐enriched (>70%) samples using a Trizol (Thermo Fisher Scientific, Carlsbad, CA, USA) method in combination with an RNeasy kit and DNase treatment, conducted following the manufacturer's instructions (Qiagen, Valencia, CA, USA). The RNA was quantified using an ND‐1000 spectrophotometer (Thermo Fisher Scientific), and its integrity was determined using an Agilent 2100 Bioanalyzer (Agilent, Santa Clara, CA, USA). The relative levels of *CLDN15* and *VIM* mRNA (primers: CLDN15‐F: ACTCCCTGGGCGTCTACAAC; CLDN15‐R: ATGGCGGTGATCATGAGTG; VIM‐F: GACAACCTGGCCGAGGAC; VIM‐R: AAGATTGCAGGGTGTTTTCG) were used to calculate the C/V score.

### Functional enrichment analysis

Differential expression analysis was performed using edgeR [34] and the raw counts of 128/174 previously published tumor RNA‐seq profiles associated with single‐pattern BAP1 staining. Specifically, pair‐wise differential expression comparisons among tumors with single‐pattern nuclear, single‐pattern cytoplasmic, and single‐pattern absent BAP1 staining were performed using a generalized linear model and quasi‐likelihood tests [35], as described in the edgeR manual. A total 17 591 genes with at least four TMM‐normalized fragments per kilobase expressed in a minimum of eight samples were considered for differential expression [36]. False discovery rate (FDR) less than 0.05 (for significance) and absolute fold‐change greater than 4 (to detect top hits) were used as thresholds to define differentially expressed genes in each comparison. Hallmark gene sets were retrieved from the Molecular Signatures Database (MSigDB: https://www.gsea-msigdb.org/gsea/index.jsp), and the fgsea (Bioconductor, https://bioconductor.org/) package was used for gene set enrichment analysis (GSEA).

### Statistical analysis

Statistical analysis and graphing were performed using Stata (StataCorp, College Station, TX, USA; Version 15.1). Fisher's exact test was used to assess associations between the presence of individual BAP1 IHC patterns and clinicopathological characteristics in categorical format or *BAP1* mutations. The Fisher–Freeman–Halton extension was used to assess the association between BAP1 staining pattern and mutations in specific protein domains and/or mutation type with the *P* value computed by the Monte Carlo method. Distributions of age, gene expression level, and expression ratio were compared among the staining patterns using a Wilcoxon rank‐sum test. The Cochran–Armitage test was used to assess the trend in frequency of staining patterns across the histologic spectrum. McNemar's test was used to compare nuclear staining between histologic components in biphasic cases. Overall survival was estimated using the Kaplan–Meier method and differences were assessed using the Cox proportional hazards model. All *P* values were based on a two‐sided hypothesis.

## Results

**Figure 1:**
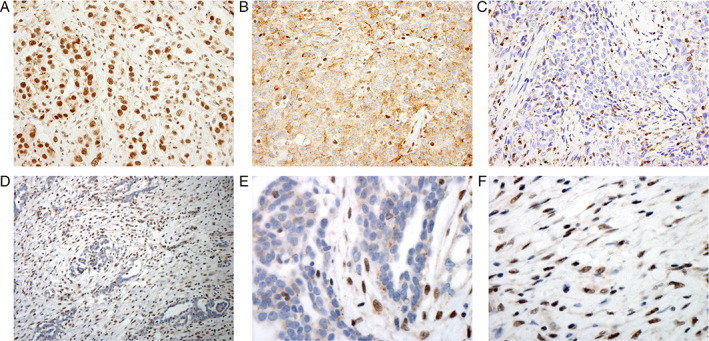
Representative images of BAP1 immunohistochemistry in MPM samples. (A) Epithelioid case displaying single‐pattern nuclear BAP1 staining; (B) epithelioid case displaying single‐pattern cytoplasmic BAP1 staining; (C) epithelioid case displaying absence of BAP1 staining; (D) biphasic case displaying absent/nuclear/cytoplasmic staining with (E) magnification (600×) of the epithelioid component with loss of nuclear BAP1 staining, and (F) magnification (600×) of the sarcomatoid component with nuclear BAP1 staining.

We analyzed slides representing 596 patients (Table 1). Representative images of BAP1 IHC staining are shown in Figure 1. Single‐pattern nuclear staining was observed in 215 (36%) tumors, single‐pattern cytoplasmic in 147 (25%), and absence of any cellular staining of tumor cells (single‐pattern absent) in 73 (12%). Combinations of staining patterns were observed in 161 (27%) tumors. The most common combination was absent/cytoplasmic (95/161; 59%). The remaining combinations, absent/nuclear (19/161; 12%), nuclear/cytoplasmic (30/161; 19%), and absent/nuclear/cytoplasmic (17/161; 11%), were analyzed descriptively because they were not observed at frequencies sufficient to support statistical comparison. Supplementary material, Tables S4 and S5 present associations of observed BAP1 staining patterns with patient clinicopathological characteristics. No associations with staining patterns were observed when subgrouping patients by self‐reported asbestos exposure or preoperative administration of neoadjuvant chemotherapy. Single‐pattern nuclear staining was higher among males (*p* = 0.026), combination absent/cytoplasmic staining was more common among females (*p* < 0.001), and single‐pattern absence of BAP1 staining was associated with younger age (*p* = 0.024).

**Table 1:** Clinicopathologic characteristics of patients included in the study.

	Patients evaluated by BAP1 IHC
Evaluable for analysis	596
Alive at last follow‐up	74 (12.4%)
Follow‐up from surgery, months	
Median (range)	34.3 (0.4–171)
Age, years	
Median (range)	66 (18–86)
Sex	
Male	439 (74%)
Female	157 (26%)
Histologic subtype	
Epithelioid	342 (57%)
Biphasic	187 (31%)
Sarcomatoid/desmoplastic	67 (11%)
Asbestos exposure self‐report	
Yes	302 (67%)
No	152 (26%)
Unknown	42 (7%)
Neoadjuvant therapy	
Yes	95 (16%)
No	487 (82%)
Unknown	14 (2%)
Single nucleotide/small variants (*N*)	263
Present	101 (38%)
Absent	162 (62%)
Copy number variation (*N*)	174
Deletion	39 (22%)
No deletion	135 (78%)
Gene expression analysis (*N*)	174

### Loss of BAP1 nuclear staining is significantly associated with SNSV mutation and large deletion

Among 263 cases with sequencing data, 113 SNSVs were observed among 101 patients (supplementary material, Table S1). Most of the mutations (89/113; 79%) caused a frameshift (*n* = 55), generated an early stop codon (*n* = 18) or altered a splice site (*n* = 16). SNSV *BAP1* mutations were significantly associated with single‐pattern absence of BAP1 staining (*p* = 0.006), and combination absent/cytoplasmic staining (*p* = 0.044) (supplementary material, Table S4). Single‐pattern nuclear staining was associated with absence of SNSV *BAP1* mutation (*p* < 0.001), whereas single‐pattern cytoplasmic staining was found in SNSV mutated and non‐mutated samples in similar proportions (supplementary material, Table S4). Among 174 cases with *BAP1* copy number alteration data, the only significant association observed was between single‐pattern nuclear staining and absence of *BAP1* deletions (*p* = 0.001).

### Cytoplasmic BAP1 staining is not associated with mutation in specific protein domains

Sixty‐five of 113 cases with SNSV had mutations in the UCH domain; the remaining mutations were distributed across the rest of the protein (supplementary material, Figure S1). No associations between BAP1 staining pattern and mutationsin specific protein domains including UCH and NLS2 and/or mutation type were observed (*p* = 0.21). Four cases exhibited mutations specifically in the NLS1 domain, of which three demonstrated cytoplasmic and one nuclear BAP1 staining.

### BAP1 staining patterns are associated with histological subtype

**Figure 2:**
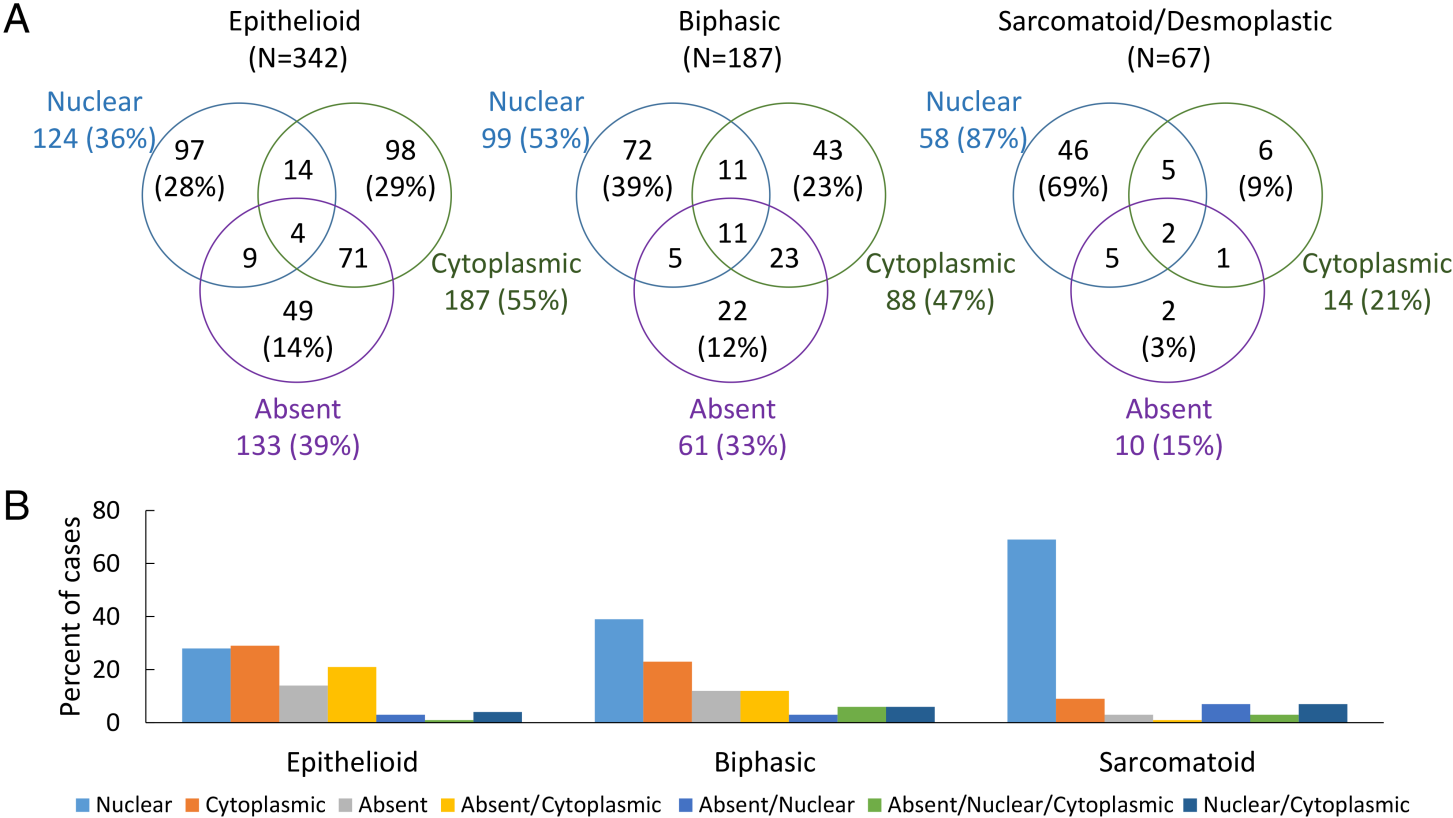
Distribution of BAP1 staining patterns in MPM subtypes. (A) Venn diagrams depicting the relation between BAP1 staining patterns and the major histological subgroups of MPM. (B) Bar charts illustrating proportions of cases demonstrating each staining pattern within histological MPM subgroups.

The distribution of BAP1 staining patterns observed among tumor histological subtypes is shown in Figure 2A. The frequency of single‐pattern nuclear staining increased across the histologic spectrum from epithelioid to sarcomatoid (*p* < 0.001 for trend). Correspondingly, the prevalence of single‐pattern cytoplasmic (*p* < 0.001), single‐pattern absent (*p* = 0.018), and combination absent/cytoplasmic staining (*p* < 0.001) decreased across the spectrum from epithelioid to sarcomatoid (Figure 2B and supplementary material, Table S4).

Fifty biphasic cases showed combination staining. In 17/50 (34%), the epithelioid and sarcomatoid components each exhibited a different staining pattern (supplementary material, Table S6). Single‐pattern nuclear staining was significantly more common within the sarcomatoid component (14/17 cases) than within the epithelioid component (4/17 cases) (*p* = 0.002). Single‐pattern nuclear staining was observed within the epithelioid component only when the sarcomatoid component also showed single‐pattern nuclear staining.

Consistent with the observed associations of nuclear staining with sarcomatoid histology and wild‐type *BAP1*, sarcomatoid samples had significantly fewer *BAP1* mutations compared with epithelioid and biphasic samples (*p* = 0.003). Sixty‐two of 146 (42%) epithelioid, 35 of 85 (41%) biphasic, and 4 of 32 (13%) sarcomatoid/desmoplastic samples showed at least one SNSV.

### BAP1 staining patterns are associated with RNA expression levels and molecular clusters

**Figure 3:**
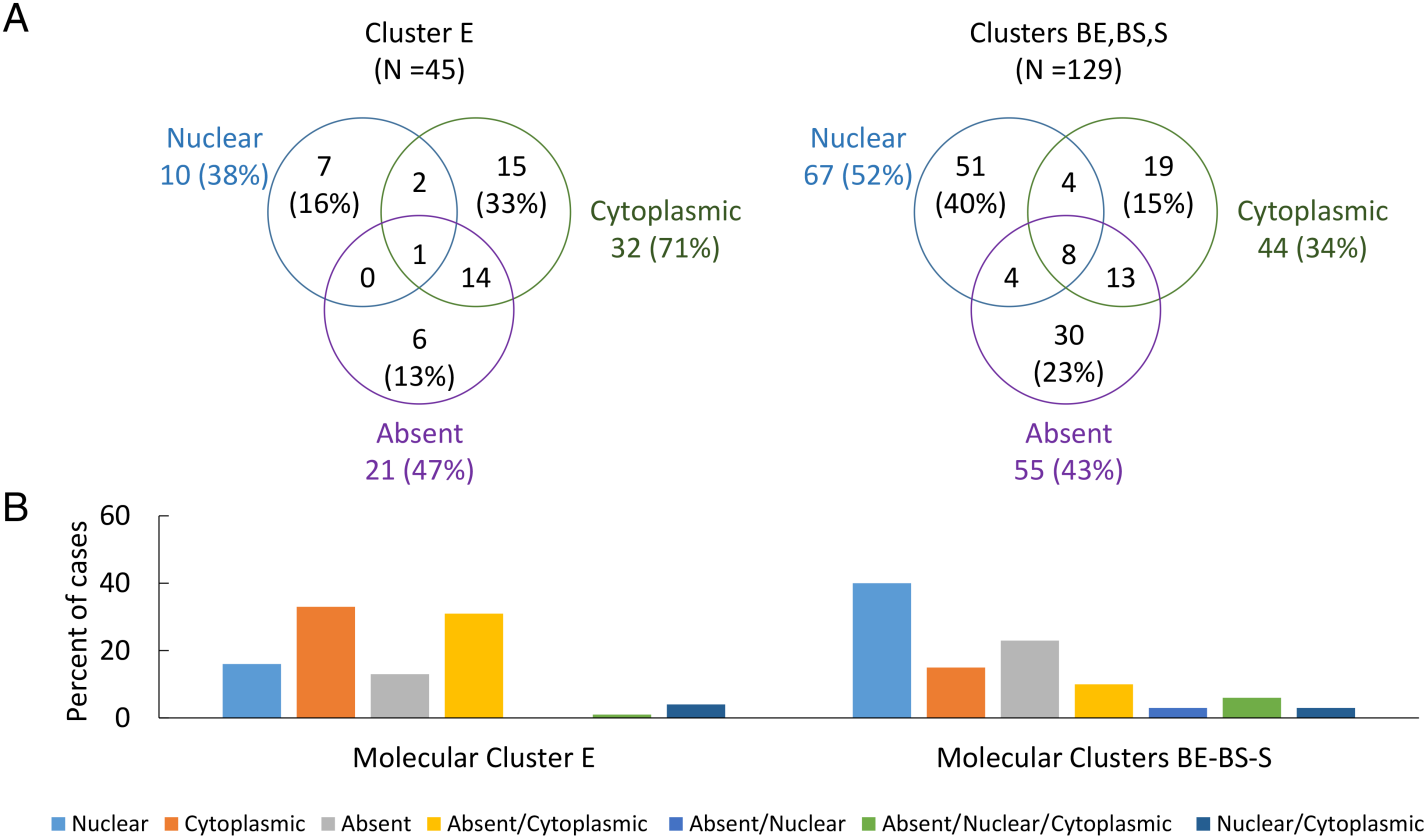
Distribution of BAP1 staining patterns among MPM molecular clusters. (A) Venn diagrams depicting the frequency of observed BAP1 staining patterns in cluster E relative to the combined remaining clusters (BE, BS, S). (B) Bar charts illustrating proportions of cases demonstrating each staining pattern within cluster E relative to the combined remaining clusters (BE, BS, S).

RNAseq and gene expression‐based unsupervised clustering analyses were available for 174/596 cases (supplementary material, Table S2) [4]. In this sub‐cohort, non‐epithelioid histology (*p* = 0.021) and single‐pattern nuclear staining (*p* < 0.001) were significantly associated with higher *BAP1* gene expression, whereas single‐pattern cytoplasmic (*p* = 0.005), single‐pattern absent (*p* < 0.001), and combination absent/cytoplasmic staining (*p* = 0.012) were associated with lower *BAP1* expression. Four molecular clusters (E, BE, BS, S) related to the spectrum from epithelioid to sarcomatoid histology were previously identified [4]. Comparison of cluster E with clusters BE, BS, and S (combined) in this subset demonstrated that the proportions of single‐pattern cytoplasmic staining (E: 15/45, 33%; others: 19/129, 15%; *p* = 0.009) and of combination absent/cytoplasmic staining (E: 14/45, 31%; others: 13/129, 10%; *p* = 0.002) were significantly higher in cluster E, whereas single‐pattern nuclear staining was more common in clusters EB, SB, and S (others: 51/129, 40%; E: 7/45, 16%; *p* = 0.003) (Figure 3A,B).

### BAP1 staining patterns are related to epithelial‐to‐mesenchymal transition (EMT) status

**Figure 4:**
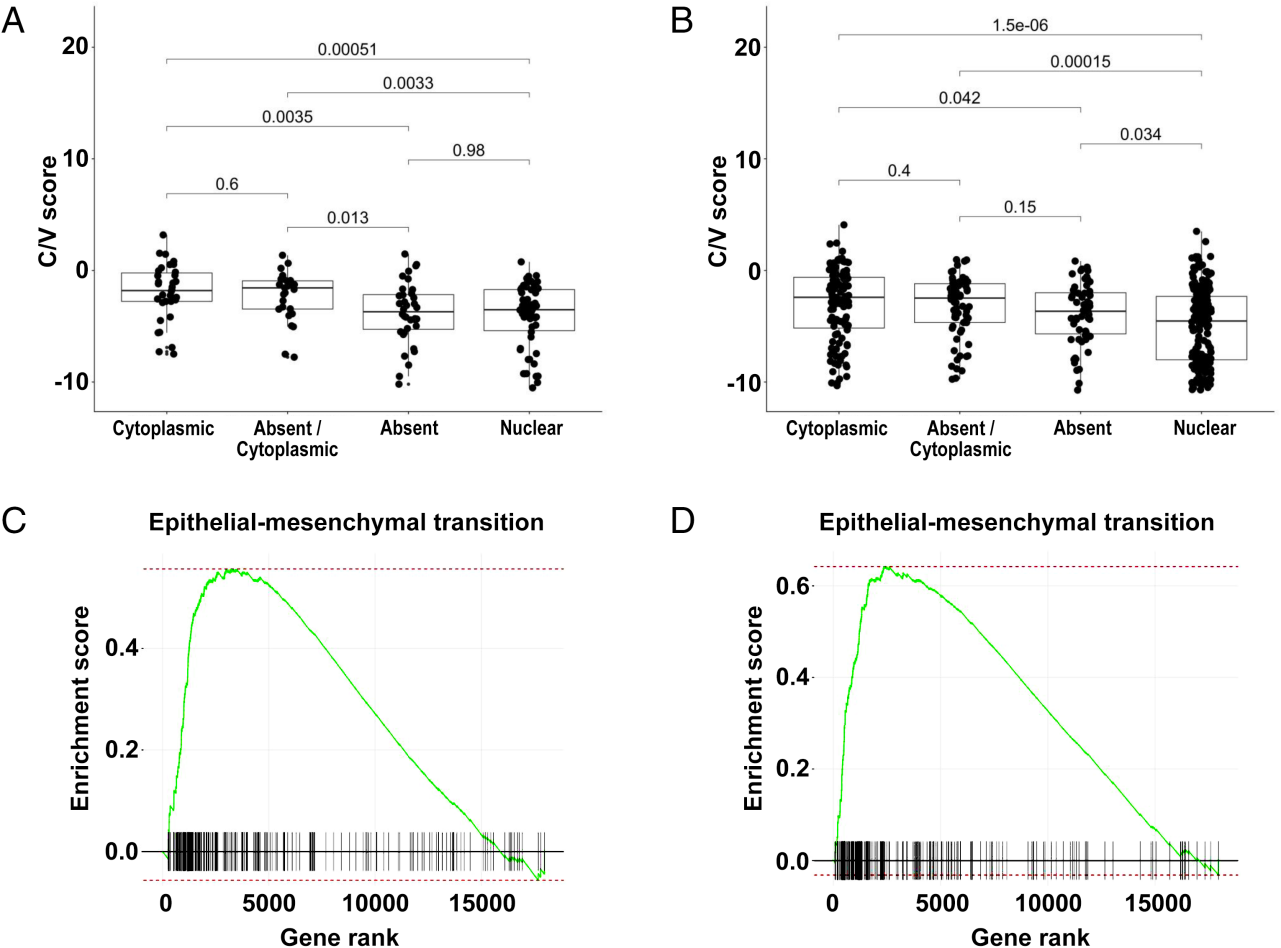
Association of BAP1 staining patterns with C/V scores and gene set expression analysis for EMT pathway. Boxplots displaying the distributions of C/V scores in relation to single‐pattern nuclear BAP1 staining, single‐pattern cytoplasmic BAP1 staining, single‐pattern absence of BAP1 staining, and combination absent/cytoplasmic staining determined using (A) RNAseq data from 155 specimens and (B) RT‐PCR data from 483 specimens. GSEA enrichment plots from RNAseq data for genes associated with the EMT process in (C) the single‐pattern nuclear BAP1 staining versus single‐pattern cytoplasmic BAP1 staining and (D) the single‐pattern absent staining versus single‐pattern cytoplasmic BAP1 staining.

In the prior study, differential expression analysis revealed that gene expression in the four clusters was related to the EMT spectrum, and that the C/V score significantly differentiates the four clusters [4]. Recently, the association of the C/V score with the EMT spectrum has been further validated [37]. We analyzed the distribution of C/V scores in relation to BAP1 staining patterns in the 155 samples with RNA‐sequencing data exhibiting single‐pattern nuclear, single‐pattern cytoplasmic, single‐pattern absent, and combination absent/cytoplasmic staining. We found that the C/V scores of samples with single‐pattern cytoplasmic and combination absent/cytoplasmic staining were significantly associated with the epithelial part of the EMT spectrum (Figure 4A). Using RT‐qPCR, the C/V scores were calculated for 483 of 596 samples, and a similar distribution of staining patterns was observed (Figure 4B). Pairwise differential expression analysis was performed among tumors displaying single‐pattern nuclear, single‐pattern cytoplasmic or single‐pattern absence of staining (128/174 samples with available gene expression profiles). Of the three possible comparisons, single‐pattern nuclear versus single‐pattern cytoplasmic, single‐pattern absent versus single‐pattern nuclear, and single‐pattern absent versus single‐pattern cytoplasmic (supplementary material, Figure S2), single‐pattern nuclear versus single‐pattern cytoplasmic showed the highest number of significant differentially expressed top hit genes (61%; supplementary material, Table S7). GSEA identified pathways with significant enrichment (supplementary material, Table S8) in all three analyses. The EMT hallmark gene set was the most significantly enriched when comparing single‐pattern cytoplasmic with single‐pattern nuclear staining (*p* < 0.001) (Figure 4C), and the second most significant comparing single‐pattern cytoplasmic with single‐pattern absent BAP1 staining (*p* < 0.001) (Figure 4D).

**Table 2:** BAP1 staining patterns, frequencies, and associations with molecular and clinical factors.

Staining pattern	BAP1 staining	Frequency	Significant association with
Single‐pattern nuclear	Nuclear localization only	36%	Male sex; sarcomatoid histology; absence of SNVS; absence of deletions; higher *BAP1* gene expression; molecular clusters BE, BS, S; lower BAP1 C/V score
Single‐pattern cytoplasmic	Cytoplasmic localization only	25%	Epithelioid histology; lower *BAP1* gene expression; molecular cluster E; higher BAP1 C/V score; better prognosis in non‐epithelioid histology
Single‐pattern absent	Absent (nuclear and cytoplasmic) staining	12%	Younger age, epithelioid and biphasic histology; presence of SNVS; lower *BAP1* gene expression
Combination of absent/cytoplasmic staining	Concurrent presence of tumor cells with complete absence of staining and tumor cells with cytoplasmic localization only	16%	Female sex; epithelioid histology; presence of SNVS; lower *BAP1* gene expression; molecular cluster E; higher BAP1 C/V score
Combination of absent/nuclear staining	Concurrent presence of tumor cells with complete absence of staining and tumor cells with nuclear localization only	3%	Insufficient frequencies for statistical comparison
Combination of nuclear/cytoplasmic staining	Concurrent presence of tumor cells with nuclear localization only and tumor cells with cytoplasmic localization only	5%	Insufficient frequencies for statistical comparison
Combination of absent/nuclear/cytoplasmic	Concurrent presence of tumor cells with complete absence of staining, tumor cells with nuclear localization only and tumor cells with cytoplasmic localization only	3%	Insufficient frequencies for statistical comparison

### BAP1 staining patterns stratify survival within non‐epithelioid histological subtypes

**Figure 5:**
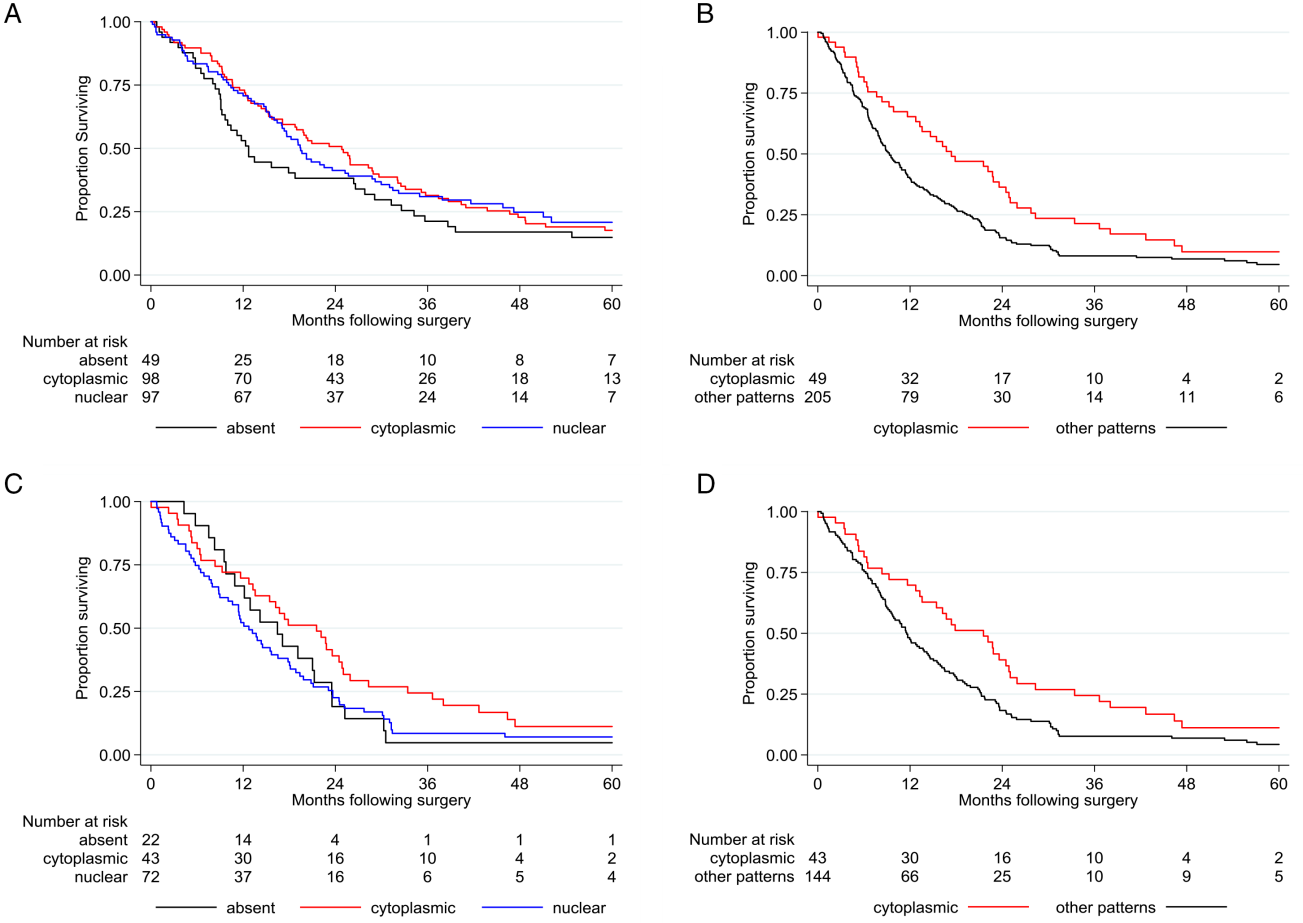
Analysis of survival in patients with MPM according to BAP1 staining pattern. Kaplan–Meier curves of patients with (A) epithelioid MPM tumors showing single‐pattern nuclear (median overall survival 19.5 months), single‐pattern cytoplasmic (median overall survival 24.8 months), and single‐pattern absent BAP1 staining (median overall survival 12.7 months); (B) non‐epithelioid MPM tumors showing single‐pattern cytoplasmic (median overall survival 17.4 months) versus other BAP1 staining patterns (median overall survival 9.3 months), *p* = 0.005; (C) biphasic MPM tumor showing single‐pattern nuclear (median overall survival 12.7 months), single‐pattern cytoplasmic (median overall survival 21.6 months), and single‐pattern absent BAP1 staining (median overall survival 16.5 months); and (D) biphasic MPM tumor showing single‐pattern cytoplasmic (median overall survival 21.6 months) versus other BAP1 staining patterns (median overall survival 11.6 months), *p* = 0.009. Overall survival was calculated from the date of surgery. Survival curves were truncated at 60 months.

Survival analysis did not reveal any significant association between BAP1 staining patterns and survival of epithelioid patients (Figure 5A), although single‐pattern absent staining trends to shorter survival (HR = 1.31 versus single pattern nuclear; *p* = 0.167). By contrast, among patients with non‐epithelioid tumors, those demonstrating single‐pattern cytoplasmic staining had a significantly longer survival than those showing other staining patterns (median 17.4 versus 9.3 months, HR = 0.62, *p* = 0.005) (Figure 5B). A similar relationship of single‐pattern cytoplasmic staining to survival was evident when biphasic tumors were considered separately (median 21.6 versus 11.6 months, HR = 0.62, *p* = 0.009) (Figure 5C,D). Because most of the sarcomatoid tumors demonstrated single‐pattern nuclear staining (69%), there was insufficient power for separate analysis. The high frequency of cytoplasmic BAP1 staining, in addition to its significant association with *BAP1* gene expression, EMT, histology, and prognosis, is inconsistent with classification as non‐specific reaction of the antibody.

No difference in patient survival was observed between cases with wild‐type versus mutated *BAP1* in this investigation (*p* = 0.58) (supplementary material, Figure S3).

Significant associations of observed BAP1 staining patterns with clinical, pathological, molecular and patient outcome variables are summarized in Table 2.

## Discussion

*BAP1* is one of the most commonly mutated genes in MPM [4]. Thus, BAP1 IHC has become part of the standard panel for the differential diagnosis of MPM. Loss of BAP1 nuclear staining is considered a reliable indicator of malignancy, particularly for epithelioid histology [21, 24]. Consistent with this diagnostic role of BAP1, we report here the increased presence of single‐pattern cytoplasmic BAP1 staining, gene mutation, and low expression in epithelioid, relative to non‐epithelioid, MPM. The predominance in sarcomatoid MPM of BAP1 single‐pattern nuclear protein localization and wild‐type genetic sequence was validated in the current study. The primary clinical impact of the current work, however, lies in the recognition that single‐pattern cytoplasmic BAP1 staining, in addition to being diagnostic of malignancy, is a putative novel prognostic biomarker identifying a subset of MPM patients with non‐epithelioid tumors who have longer survival after surgical resection.

Previous studies have suggested that specific mutations of *BAP1* in the UCH and NLS2 domains may be associated with cytoplasmic staining, supporting the hypothesis that these mutations determine cytoplasmic BAP1 protein localization [6, 17, 28, 29, 38]. In the present study, three mutations were observed in NLS1 associated with cytoplasmic BAP1 staining and none in NLS2, representing only 1% of the 289 cases with observed cytoplasmic staining. No association between single‐pattern cytoplasmic BAP1 staining and specific *BAP1* mutations in UCH or any other domain of the protein was observed. This observation may be explained by the fact that the antibody binds the C‐terminal region of the protein. Because most of the mutations in *BAP1* cause a frameshift or early stop codon within the transcript [3, 4, 5, 17, 39], this antibody would be unlikely to bind many of the mutated forms of *BAP1*, if translated, suggesting that observed cytoplasmic staining represents binding of full‐length or nearly full‐length protein translated from the other (wild‐type) allele. This interpretation suggests a gene‐dosage effect, consistent with the observed significant association of single‐pattern cytoplasmic staining with low RNA expression [40]. Therefore, cytoplasmic staining may represent translation of one wild‐type allele, whereas nuclear staining may require translation of both wild‐type alleles.

Current clinical use of BAP1 IHC involves binary evaluation of presence versus loss of nuclear staining [18, 19, 20, 21, 22, 23, 25, 26, 27]. Unexpectedly, only half of tumors displayed single‐pattern absent (12%) or single‐pattern nuclear BAP1 staining (36%). The remaining samples had single‐pattern cytoplasmic (25%) or combinations of nuclear, cytoplasmic, and/or absent BAP1 staining (27%). Single‐pattern absent, single‐pattern cytoplasmic, and combination absent/cytoplasmic staining were predominantly associated with epithelioid and biphasic histology tumors. In addition, 17/186 (9%) biphasic cases showed different BAP1 staining in their histological compartments, as observed by others [9, 10, 13, 23, 25], underscoring the association of BAP1 protein localization with histology, and consistent with the hypothesis that biphasic mesothelioma represents genomic heterogeneity of *BAP1*. The high frequency of single‐pattern cytoplasmic staining, occurring in 25% of the samples, and its association with a subgroup of patients with favorable prognosis, observed in this study, argue against the interpretation of cytoplasmic BAP1 as non‐specific antibody staining, suggesting instead that BAP1 protein is frequently localized in the cytoplasm [17, 18, 38]. Consistent with this interpretation, a functional role of cytoplasmic BAP1 has recently been proposed, modulating calcium release from the endoplasmic reticulum into the cytosol and mitochondria, and promoting apoptosis [41].

The association of MPM with the EMT spectrum has long been recognized [42]. EMT is a reversible cellular process that transiently places epithelial cells into a quasi‐mesenchymal cell state displaying multiple traits associated with high‐grade malignancy [43]. We show here that single‐pattern cytoplasmic BAP1 staining is associated with lower *BAP1* gene expression, epithelial‐like EMT markers, epithelioid tumor histology, and longer patient survival. These findings are supported in MPM by *in vitro* studies [44], and by a recent investigation in clear cell renal cell carcinoma patients in which lower *BAP1* expression was correlated with longer overall survival [45]. In the same study, inactivation of *BAP1* in clear cell renal cell carcinoma cell lines altered cellular proteostasis, suppressed cell proliferation, and induced a mesenchymal‐to‐epithelial‐like phenotype [45], suggesting that the role of BAP1 in tumor progression may be more complex than its presumed tumor suppressor function [46]. In support of this interpretation, recent findings reveal that low *BAP1* expression is associated with longer survival in cutaneous melanoma, but shorter survival in uveal melanoma [47]. A putative role of BAP1 in the underlying biology of EMT in MPM is intriguing and the current data encourage further exploration.

Some studies have shown that a subset of patients with non‐epithelioid MPM who undergo surgery can have an extended survival [48, 49, 50]. Unfortunately, no biomarker is available to identify these patients. Many solid tumors, such as non‐small cell lung cancer, are driven by specific mutations in oncogenes, leading to the development and successful use of targeted sequencing panels to select targeted therapies and predict survival of cancer patients [51]. MPM, by contrast, has been characterized primarily by tumor suppressor loss, and in the case of *BAP1*, by mutations in tumor cells that are highly variable among tumors and thus less practical to detect using a targeted sequencing approach. Furthermore, our data based on exome sequencing of the entire *BAP1* gene do not reveal differential patient prognosis between cases with wild‐type versus mutated *BAP1*. Currently, routine BAP1 IHC is performed to assist differential diagnosis of MPM, inferring the presence of *BAP1* mutation, deletion or silencing based on loss of BAP1 nuclear staining. Our finding that single‐pattern cytoplasmic BAP1 is associated with improved survival of patients with non‐epithelioid MPM suggests that BAP1 IHC may also be useful as a prognostic biomarker, as has been suggested for glioma [52]. If validated, BAP1 IHC would provide an urgently needed and clinically relevant biomarker for the stratification of patients with biphasic MPM into risk groups to guide treatment decisions and protocol enrollment [48].

Recent investigations in peritoneal mesothelioma have suggested that *BAP1* haploinsufficiency is correlated with an inflammatory tumor microenvironment and may be a potential prognostic and predictive biomarker for immunotherapy [53]. In addition, reduced *BAP1* RNA expression and protein nuclear loss have been correlated with immune modulation and poor outcomes in primary and metastatic uveal melanoma [54]. We conducted two exploratory analyses to evaluate the expression of immune genes in high versus low *BAP1* expression (either below and above the median *BAP1* expression level, or between the first and fourth quartiles of expression) using RNAseq data from 174 MPM tumors. The ‘interferon gamma response’ gene set was significantly enriched in high *BAP1* compared with low *BAP1* non‐epithelioid tumors (*p* = 0.03 and *p* = 0.02 for high versus low and first versus fourth quartiles, respectively) (first versus fourth quartile analysis, supplementary material, Figure S4A), whereas the ‘interferon alpha response’ gene set was significantly enriched only in the first to fourth quartile comparison (*p* = 0.01; supplementary material, Figure S4B).

Our study has several limitations. First, heterogeneous staining was common even within single tissue sections, suggesting that additional slides may be required to assess whether distinct morphological, genetic, and phenotypic profiles exist in other areas of the tumor. Undetected heterogeneity may have resulted in mismatch between mutational analysis, performed on frozen tissue samples, and clinical FFPE blocks used for immunohistochemistry. Second, only mutational data from deep sequencing were considered; additional analyses may be necessary to comprehensively identify all relevant genetic *BAP1* alterations [17]. Finally, large epigenetic analyses of the *BAP1* promoter have not been performed for MPM samples; therefore, the possibility that epigenetic mechanisms may contribute to loss of nuclear *BAP1* staining in cases where gene mutation is not evident cannot be eliminated.

In conclusion, our findings indicate that the subcellular localization of BAP1 within the nuclear and/or cytoplasmic compartment, or its absence, is associated with clinical, histologic, and molecular features. Combinations of BAP1 staining patterns are frequent in MPM, suggesting that careful analyses in adequate specimens should be performed to evaluate BAP1 staining and avoid sampling bias. Single‐pattern nuclear staining is significantly associated with wild‐type *BAP1* and sarcomatoid subtype. We identified an association of single‐pattern cytoplasmic staining with markers of EMT, suggesting a complex role for BAP1 in MPM. Finally, this investigation has identified single‐pattern cytoplasmic staining as a putative indicator of favorable prognosis, providing a practical biomarker that addresses a critical need in clinical decision‐making for patients with non‐epithelioid MPM.

## Author contributions statement

ADR, RB and WGR conceived and designed the study. ADR and WGR developed the methodology. LRC and YPH carried out histological analysis. DTS, SF and RVJ carried out bioinformatic analysis. BYY and WGR performed statistical analysis. NTD, CVM and MEO were responsible for RNA extraction. ADR, BYY, RB and WGR wrote, reviewed, and/or revised the manuscript. CEG provided administrative, technical, or material support. RB and WGR had final approval of the article.

## Supporting information


**Figure S1.** Schematic representation of 113 SNSV *BAP1* mutations identified in 101 patients with MPM
**Figure S2.** Differential expression analysis of 128 MPM tumors with single‐pattern BAP1 staining
**Figure S3.** Survival analysis of 199 patients with MPM carrying wild‐type or mutated *BAP1*
**Figure S4.** Gene set expression analysis for exploratory analyses of high versus low *BAP1* expression

**Table S1.** *BAP1* mutation details among 263 samples with available sequencing data
**Table S2.** *BAP1* RNA expression data in 174 MPM samples
**Table S3.** Schematic representation of the overlap of samples among molecular analyses
**Table S4.** BAP1 staining pattern in relation to the patients' characteristics
**Table S5.** *P* value of the distribution of the BAP1 staining patterns in relation to the patients' age, gene expression, and C/V ratio
**Table S6.** Biphasic MPM cases with different BAP1 staining in the diverse cellular components
**Table S7.** List of significant differentially expressed top hit genes among pairwise comparisons of single‐pattern staining
**Table S8.** List of significant enriched pathways identified by gene set enrichment analysis among pairwise comparisons of single‐pattern staining
