# Supplementary material for: Large‐scale analysis of BAP1 expression reveals novel associations with clinical and molecular features of malignant pleural mesothelioma
Source: J Pathol. 2020 Oct 15;253(1):68–79. doi: 10.1002/path.5551 (PMC7756745; doi:10.1002/path.5551)
Supplement: Supplementary file 1 — Figure S1. Schematic representation of 113 SNSV BAP1 mutations identified in 101 patients with MPM Figure S2. Differential expression analysis of 128 MPM tumors with single‐pattern BAP1 staining Figure S3. Survival analysis of 199 patients with MPM carrying wild‐type or mutated BAP1 Figure S4. Gene set expression analysis for exploratory analyses of high versus low BAP1 expression [file PATH-253-68-s001.docx]

**Large-scale analysis of BAP1 expression reveals novel associations with clinical and molecular features of malignant pleural mesothelioma**

A De Rienzo *et al. J Pathol* DOI: 10.1002/path.5551

**Supplementary Figures S1–S4**


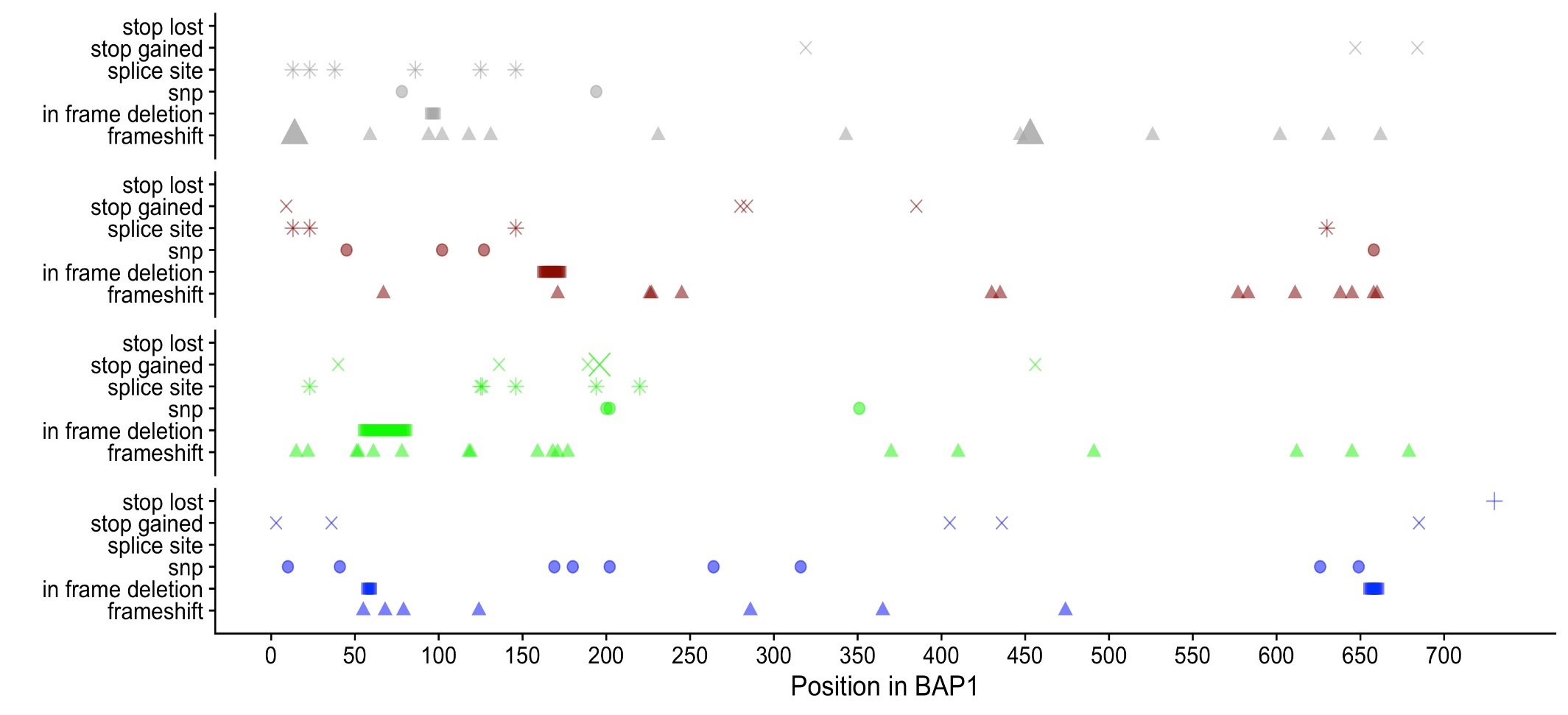

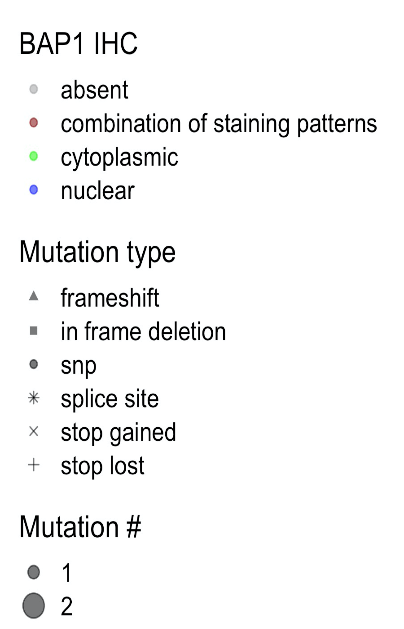


UCHR Region (1-240)

BARD1 Binding Domain (182-363)

HBM

(363-366)

BRCA1 Binding

Domain (594-657)

Putative NLS

(656-661 and 717-722)

BAP1 (C-4): sc2838 (aa 430-729)

**Figure S1. Schematic representation of 113 SNSV *BAP1* mutations identified in 101 patients with MPM.**

The position of amino acids affected by *BAP1* mutation in the BAP1 protein sequence are depicted grouped by BAP1 staining pattern. Absent BAP1 staining is shown in grey, combinations of BAP1 staining patterns in brown, cytoplasmic BAP1 staining in green, and nuclear BAP1 in blue. Different mutation types are illustrated with different symbols. The size of each symbol reflects the number of mutations in the same position.


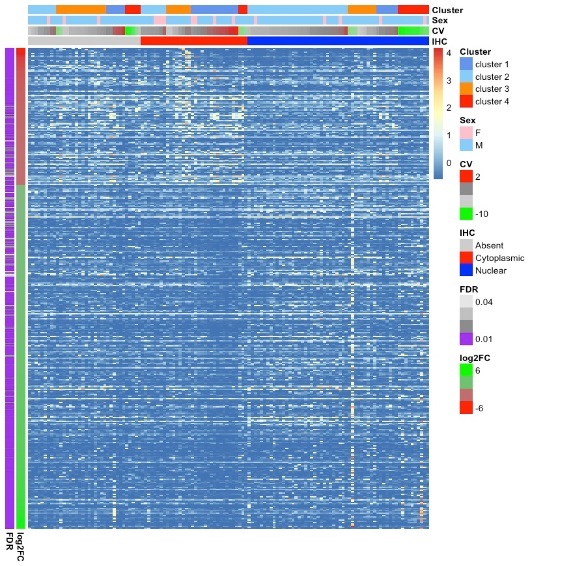


**D**


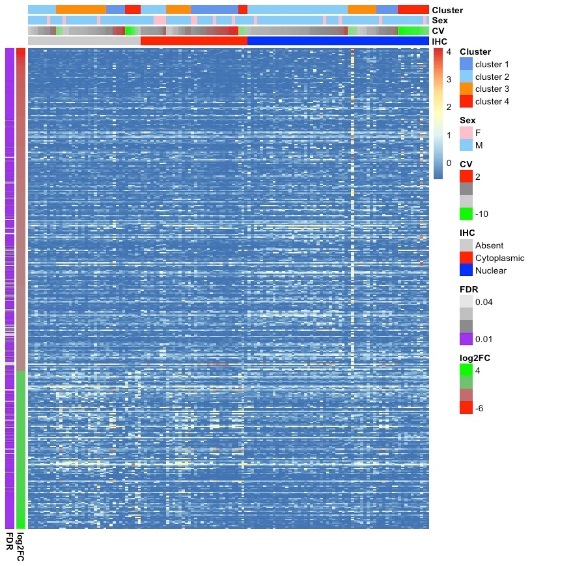


**E**


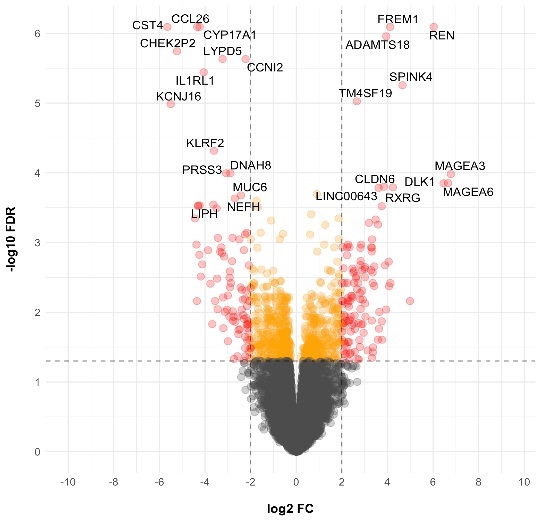


**C**

**Absent versus Cytoplasmic**

**G**


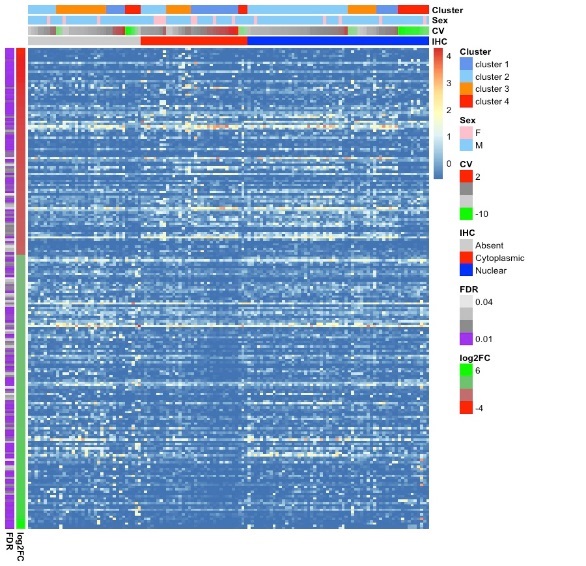


**F**

**Significant genes**

**Top hit genes**

**H**


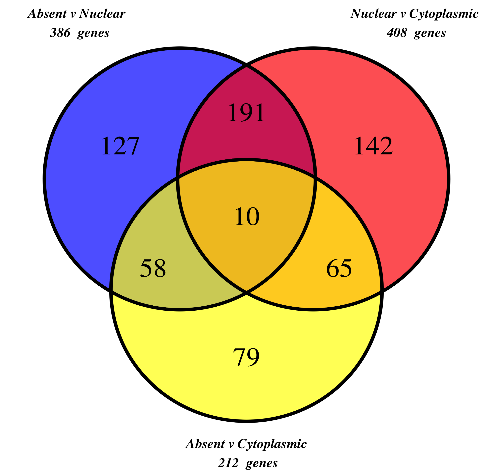

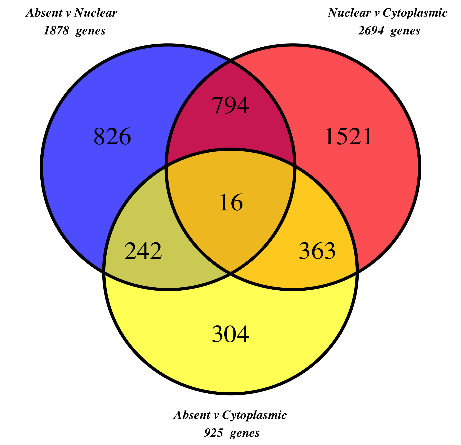


**Absent versus Nuclear**

**B**


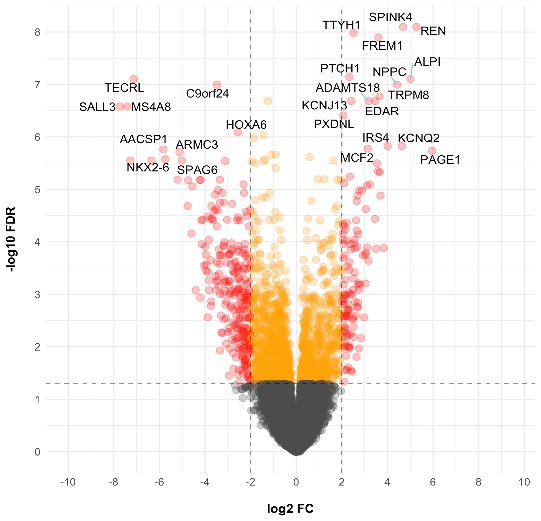


**Nuclear versus Cytoplasmic**

**A**


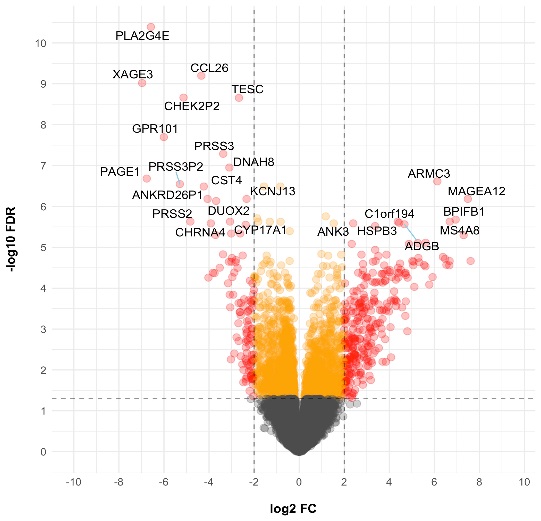


**Figure S2. Differential expression analysis of 128 MPM tumors with single-pattern BAP1 staining.** Volcano plots representing (A) differentially expressed genes between samples showing single-pattern nuclear BAP1 staining and single-pattern cytoplasmic BAP1 staining, (B) single-pattern absent BAP1 staining and single-pattern nuclear BAP1 staining, and (C) single-pattern absent BAP1 staining and single-pattern cytoplasmic BAP1 staining. The -log10 false discovery rate (FDR) was plotted against log2 fold-change (FC) values for all tested genes. The symbols on the negative and positive values of X-axis in each figure represent downregulated and upregulated genes, respectively. Symbols corresponding to significantly differentially expressed genes (FDR <0.05) and top hit genes (FDR<0.05 & 4-fold change) are colored in yellow and red, respectively. Heat maps showing the expression (log10 transcripts per million) of top hit genes differentially expressed between samples displaying (D) single-pattern nuclear BAP1 staining and single-pattern cytoplasmic BAP1 staining, (E) single-pattern absent BAP1 staining and single-pattern nuclear BAP1 staining, and (F) single-pattern absent BAP1 staining and single-pattern cytoplasmic BAP1 staining. Molecular cluster, sex, CV score and BAP1 staining pattern of tumor samples (columns) and FDR and log2 FC of genes (rows) are annotated by color. Venn diagrams showing (G) the number of differentially expressed genes and (H) the number of the top hit genes in the three comparisons.

**Figure S3. Survival analysis of 199 patients with MPM carrying wild-type or mutated *BAP1.*** Survival was estimated in 199 patients, 77 with wild-type *BAP1* sequence and no deletion detected, and 122 with at least one *BAP1* SNSV and/or deletion. Sixty-four wild-type sequenced cases were not included in the analysis because copy number information was not available. The median overall survival was 16.7 months for patients carrying *BAP1* alterations and 16.0 months for patients with wild-type *BAP1*. Overall survival was calculated from surgery. Survival curves were truncated at 60 months.


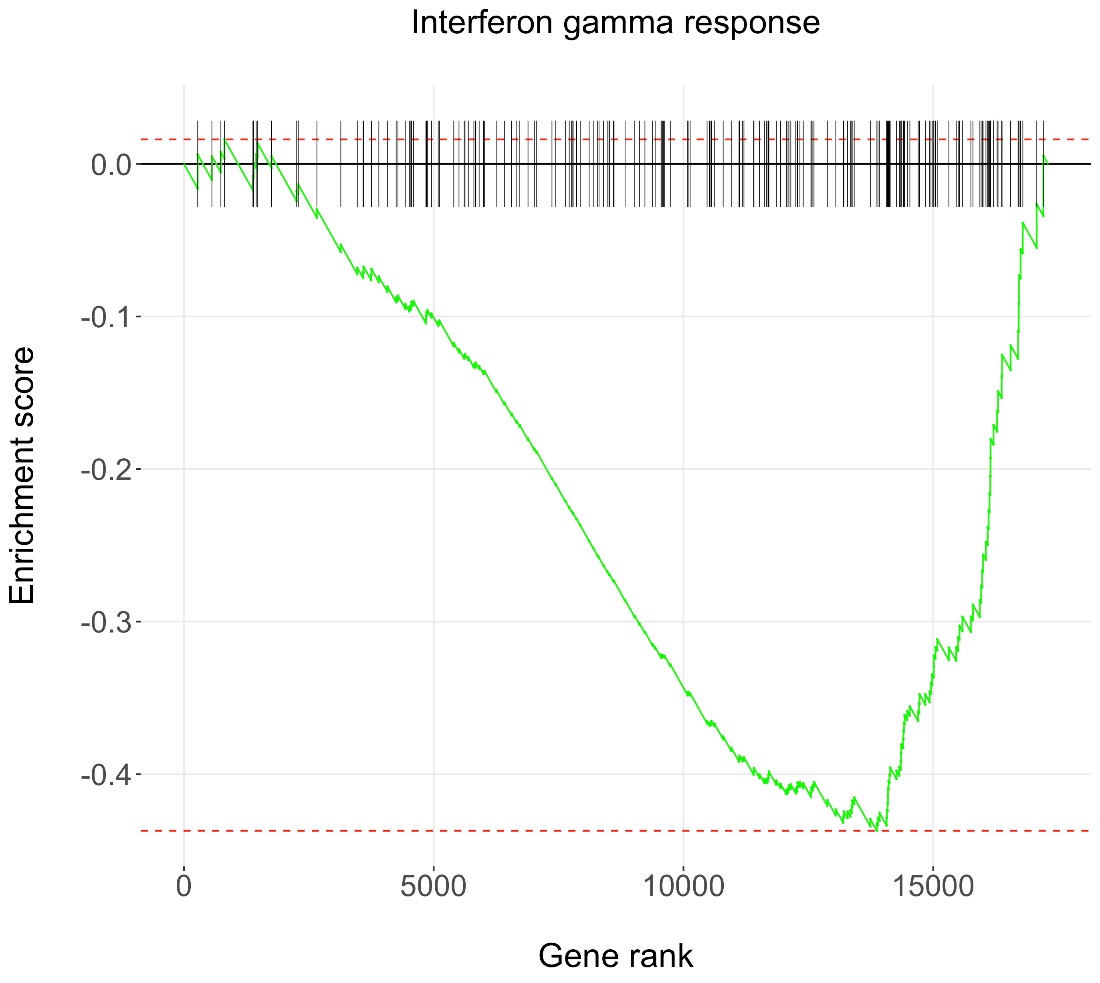


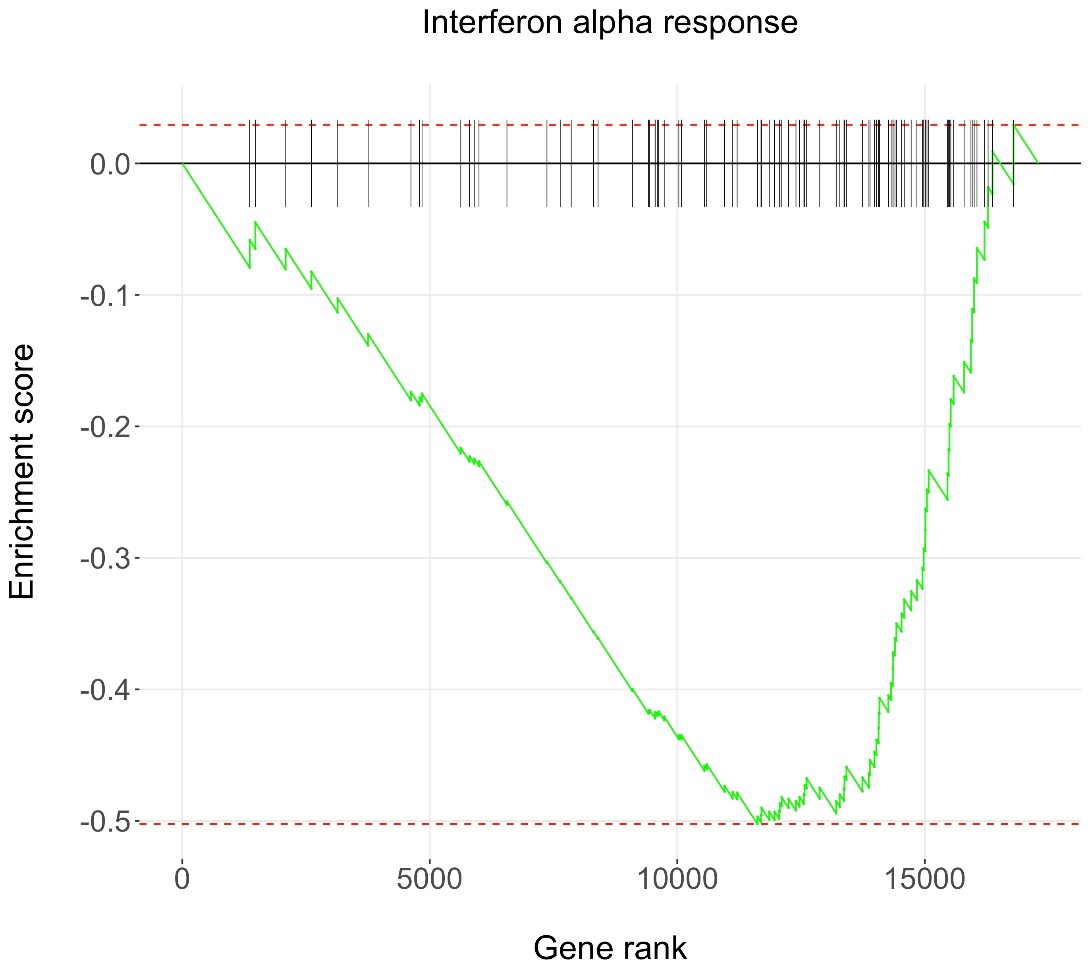


**Figure S4. Gene set expression analysis for exploratory analyses of high versus low *BAP1* expression*.*** GSEA enrichment plots from RNAseq data (A) for genes associated with the “interferon gamma response” gene set, and (B) genes associated with “interferon alpha response” gene set comparing the first to fourth quartile of expression (n = 88).
